# Effects of deliberate practice and structured feedback in psychotherapy training (DeeP): a study protocol of a randomized-control-trial

**DOI:** 10.1186/s40359-024-02015-x

**Published:** 2024-12-04

**Authors:** Anna Berning, Stefan Sell, Wiebke Andersen, Bernhard Strauß, Svenja Taubner

**Affiliations:** 1 Institute for Psychosocial Prevention, University Hospital Heidelberg, Heidelberg, Germany; 2 Psychological Institute, University Heidelberg, Heidelberg, Germany; 3 Institute for Psychosocial Medicine, Psychotherapy and Psychooncology, University Hospital Jena, Jena, Germany; 4 MAPP-Institute, Magdeburg, Germany; 5 Deutsches Zentrum für Psychische Gesundheit (DZPG), Halle-Jena-Magdeburg, Germany

**Keywords:** Interpersonal competence, Psychotherapy training, Randomized-control-trial, Therapeutic alliance, Rupture-repair

## Abstract

**Background:**

Psychotherapeutic competencies encompass a variety of skills that influence the work and therapeutic success of psychotherapists. In particular, interpersonal skills and the associated ability to react appropriately in complex therapy situations have already shown significant correlations with later therapeutic success. Strengthening interpersonal skills should therefore be a central aim of psychotherapy training. However, previous studies have shown that not only content learning is decisive for later learning success and skill development, but also the didactic method has a significant effect. Deliberate Practice and Structured Feedback have shown to be promising didactic tools. The primary objective of this study is to investigate the efficacy of Deliberate Practice and Structured Feedback as well as their combination in the context of psychotherapy training in comparison to classic didactic training. The trainees’ learning progress of interpersonal skills and its influence on subsequent outpatient therapies will be examined. Secondary, the study aims to identify further determinants of trainees’ therapeutic skills and to investigate the effects of ruptures on the course of therapy.

**Methods:**

The underlying study, a randomized-control-trial with three intervention groups (Deliberate Practice, Structured Feedback, combination of both) and an active control group, will be conducted on a sample of *N* = 240 trainees and their patients (*N* = 1000). All trainees will each attend three consecutive workshops on “Strengthening therapeutic skills in challenging therapy situations”, which will be carried out using the corresponding didactic tool. Scientific assessments will take place online for the trainees (pre, in-between, post, follow-up) and for the patients (after each therapy session for 24 weeks, follow-up). Primary outcomes will be constituted by the trainees’ Facilitative Interpersonal Skills and the patients’ symptoms. Multilevel as well as structural equation modelling will be used to analyze the data.

**Discussion:**

The study investigates the efficacy of different didactic tools regarding the strengthening of trainee’s interpersonal competencies as well as their effects on subsequent therapies. Results of this study address a research gap concerning the improvement of psychotherapy training as well as the quality assurance of future therapies.

**Trial registration:**

German Clinical Trial Register (DRKS) – DRKS00034279; retrospectively registered.

**Supplementary Information:**

The online version contains supplementary material available at 10.1186/s40359-024-02015-x.

## Background

Psychotherapy training institutions bear a crucial responsibility for the development of therapists’ competencies and serve as the first link in the chain for assuring the quality of psychotherapy. Based on the finding that therapist-specific variables (e.g., therapeutic competencies) can explain therapy-outcome variance across different types of treatment, it is evident, that competencies and skills should be trained appropriately during psychotherapy training, and that trainees should receive the best possible education [1–3]. In order to design the best possible training, two aspects should be considered: (1) What competencies are relevant with regard to psychotherapy (outcome) [4], (2) Which didactic training method is best to learn and practice competencies and hence promises the greatest learning success [5–8]. Consequently, when designing psychotherapy training research, both aspects, the targeted competencies and the training method, should be considered. Despite the importance of this topic, there is a lack of systematic studies on how specific skills can be targeted in psychotherapy training [9] resulting in a need for research on evidence-based psychotherapy training [10].

In general, competence in psychotherapists can be defined as the flexible and adequate use of therapeutic techniques. This flexibility ensures an adaptation of the interventions to the psychotherapy context as well as the patient variables and thus reflects an appropriate handling of the therapeutic situation [11]. With regard to specific competent psychotherapeutic behaviors and techniques, definitions commonly differ depending on the therapy orientation [11, 12]. Anderson’s contextual model of therapeutic skills provides a comprehensive, interdisciplinary model of competence, containing four global and interrelated domains (technical, relational, conceptual, and cultural competence) [13]. Although this model also includes therapy-school-specific interventions as a part of the technical competence, it provides three broad domains of competent behaviors that are not bound to a certain orientation. Previous research has repeatedly sought to investigate the effects of those competencies on therapy success with several studies confirming them as a significant predictor of therapy outcome [14–16]. In particular, the importance of the therapist’s relational skills for the course of therapy and its success was demonstrated in meta-analyses and experimental studies [17, 18]. Nevertheless, a generalized statement about the impact of the relational skills can only be made with caution due to the large number of different definitions and variables considered as well as the lack of standardized measures and randomized-control-trials (RCT) [17, 19]. Consequently, it seems reasonable to consider individual domains of competence in a differentiated manner and to assess them using specifically developed standardized tools. As Heinonen and Nissen-Lie [4] pointed out, the research of relational competencies, measured with performance-based tasks eliciting complex situations, displays promising results and consistent associations between the therapist’s relational skills and therapy outcome. Accordingly, the expansion of this branch of research using an RCT with standardized, performance-based measures is a promising approach to fill the given research gap.

An operationalization for the relational competence of therapists frequently used in research is Anderson’s concept of *Facilitative Interpersonal Skills* (FIS) [20]. The FIS comprise several verbal and non-verbal skills such as “sociability, empathy, and the ability to perceive, decode, and send a wide range of interpersonal communications” [21, p. 314]. To operationalize and standardize the measurement of the construct, it was divided into eight subcategories *(verbal fluency; hope and positive expectations; persuasiveness; emotional expression; warmth*,* acceptance and understanding; empathy; alliance bond capacity; alliance rupture-repair-responsiveness)*. The survey of these eight subcategories is carried out in the form of the video-based FIS performance task (further information on measurement in the methods section) [20]. In recent years, the FIS performance task has proven to be a reliable measurement tool that shows associations with therapy-relevant process variables and therapy success [22]. For instance, past research has shown that the FIS have a consistent relationship to subsequent treatment success in prospective studies [23, 24] including RCTs [25]. Hence, it appears to be advisable to target and strengthen relational competencies (in the form of the FIS) as part of psychotherapy training in order to improve the psychotherapy’s subsequent success and ensure therapy quality. However, there has not yet been an investigation of these effects in form of an RCT in Germany.

With regard to the mechanisms, by which the FIS work and thus improve the success of psychotherapy, the therapeutic alliance must be taken into consideration. Bordin defined it as the key mechanism to the change process within therapy. In the course of this he divided the alliance into 3 aspects: (1) Agreement in goals, that are to be achieved in therapy, (2) Collaboration between the patient and therapist on the tasks that contribute to the achievement of the goals, (3) Affective bond between the patient and therapist, that is characterized by respect and appreciation [26]. A comprehensive therapeutic alliance, that can be characterized as the basis for a goal-oriented collaboration within therapy, is achieved through the fulfillment of all three aspects. Longitudinal [27–29] as well as meta-analytical [30–32] research within different orientations of therapy have already pointed out the influence of alliance on psychotherapy outcome variables. Any kind of discrepancy in one of the three facets constitutes a rupture in the alliance [33]. The intensity of such a rupture can range from a minor dissonance or tension to an actual breakdown of the therapeutic relationship. Alliance ruptures can generally be organized into two categories: withdrawal and confrontation ruptures [34]. Withdrawal ruptures can be characterized by the patient withdrawing or disengaging from the therapist, their emotions or the therapeutic process itself. In case of a confrontation rupture on the other hand, the patient is moving against the therapist, e.g., by expressing dissatisfaction about the therapy progress or by pressuring the therapist [34]. Alliance ruptures can be resolved/repaired by the therapist using direct or indirect strategies (e.g., exploring the rupture or linking the rupture to interpersonal patterns). In principle, a rupture is considered repaired when the patient and therapist can resume the therapeutic work collaboratively within their affective bond [32]. Within this process the reasonable use of the FIS can help to build up the alliance, and in particular to manage possible ruptures and apply appropriate strategies [21]. With regard to the positive association between high interpersonal skills in therapists and the good handling of ruptures as well as a high therapeutic alliance there have only been a few studies to date, yet they have all been able to confirm this relationship (e.g., [25]). In particular, it should be emphasized that although the therapeutic alliance has already been confirmed as a general mediating factor in previous studies [35, 36], there is still no research on the mediating function of the alliance with regard to the relationship between FIS and the therapeutic outcome.

As a number of studies have already shown, the therapeutic alliance can be improved through targeted training (e.g., Alliance-Focused Training (AFT; [37])) [8, 38, 39]. Nevertheless, it should be noted that there are very few systematic RCTs to date that examine training on interpersonal skills and/or alliance across different psychotherapeutic orientations and also investigate the training methods used. However, as already pointed out, the aspect of the didactic tool should not be neglected when developing a training program. Irrespective of the training content, two training methods in particular have proven to be effective - Deliberate Practice and Structured Feedback. Deliberate Practice can be defined as „individualized training activities […] to improve specific aspects of an individual‘s performance through repetition and successive refinement“ [40, pp. 278–279]. The training thereby consists of a well-defined desired performance goal, a practice activity, immediate feedback on the activity as well as a repetition of the practice-feedback loop [41]. This method can be and has already been used efficiently in different fields such as sports, music, games but also the training of professionals [42]. In addition, Deliberate Practice has proven to be an effective method in psychotherapy training, as associations between Deliberate Practice and subsequent therapeutic success have been found (e.g., [43–45]). However, as Mahon noted in his scoping review, the studies often have small sample sizes and commonly do not investigate lasting effects through follow-ups. In addition, there is great heterogeneity with regard to the interventions used [46]. Furthermore, the relationship between Deliberate Practice and subsequent patient outcome has been investigated only marginally, which in turn results in a need for research [46]. The second promising didactic tool is Structured Feedback which is comprised of explicit feedback interventions that provide competency feedback using established categories, feedback schemes, or guidelines [47]. Using the interpersonal skills as an illustration, these feedback scales could, for example, be represented by the eight FIS-categories [48]. Analogously to Deliberate Practice, (Structured) Feedback has been successfully investigated in different fields such as medical training [49], sports [50] as well as psychotherapy training [7]. Nonetheless, it should also be stressed that to date there have been no longitudinal studies across psychotherapeutic orientations regarding the effects of Structured Feedback on the learning success of psychotherapy trainees and its subsequent effects on therapy success. Going one step further, it could be assumed that a combination of these promising didactic tools holds the potential for a particularly high learning success. This would involve frequent and small-scale practice with a specific goal in accordance with the principles of Deliberate Practice while the respective feedback on the practice processes would be carried out using Structured Feedback scales. A combination of these didactic methods paired with a training for interpersonal skills and investigated within an RCT that examines both the increase in skills and the effects on subsequent therapy outcomes promises to address a major research gap and training quality requirement.

## Methods

### Aims, design, and setting

The longitudinal four-arm randomized-control superiority trial will be the first study to investigate the efficacy of Deliberate Practice and Structured Feedback as well as their combination in the context of psychotherapy training. Consequently, three intervention groups as well as an active control group will be investigated in the study. The primary objective of the study is to analyze the effects of the didactic methods on the trainees’ learning progress and, in a later stage, on the trainees´ subsequent outpatient therapies. Within this process, possible mediating factors, such as the perceived therapeutic alliance, are also considered. As a secondary objective, the project aims to identify further determinants of trainees’ therapeutic skills development, to investigate the effects of ruptures on the course of therapy and to establish and validate the German version of various measures.

In addition, a qualitative part of the study intends to gain in-depth insight into the trainees’ perspective on interpersonal competencies and their development. Furthermore, its objective is to understand trainees’ experiences with and views of the training as well as the corresponding learning processes and possible barriers and facilitators of skill development.

**Fig. 1:**
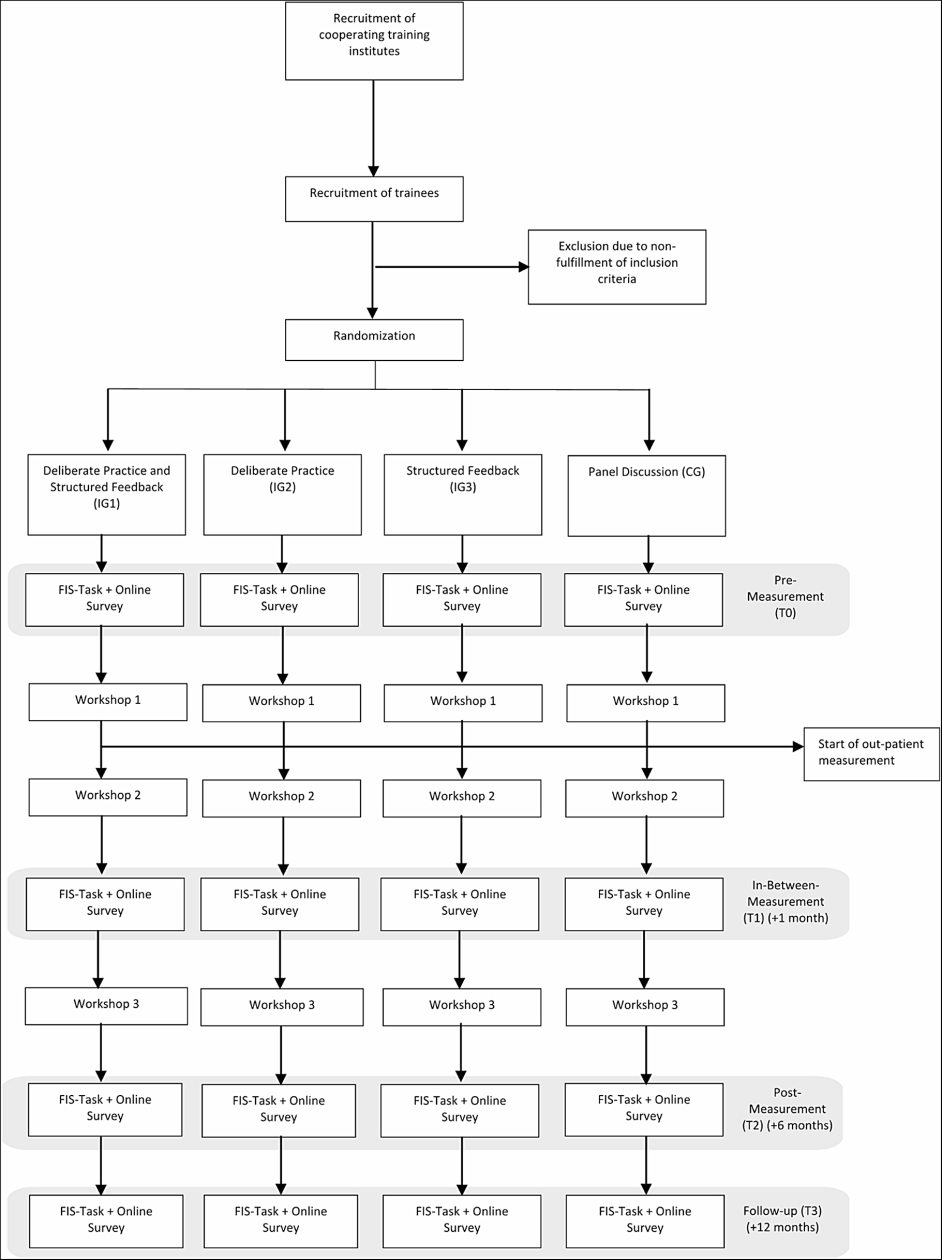
Consort diagram

Ethic approval has been obtained from the two local study centers’ ethics committees (Heidelberg, AZ Tau 2022 6/2-A3, and Jena, AZ 2023–2870_3-BO). The survey will be carried out online using SoSci Survey [51] for the assessment of questionnaires and a self-developed website for the implementation of the online FIS Performance Task. The intervention takes place in the form of a three-parts seminar, which extends over a period of six months (workshop 1: +0 months, workshop 2: +1 month, workshop 3: +6 months). The surveys take place accordingly during the pre-workshop (t0: +0 months), in-between (t1: +1 month), during the post-workshop (t2: +6 months) and as a follow-up (t3: +12 months) (see Fig. 1).

The corresponding patients of participating trainees will be surveyed for a time span of 24 weeks, starting after their therapists’ first workshop. They will complete one questionnaire per week after each therapy-session. The follow-up for the patients will take place another six months later.

### Recruitment and sample

During the initial phase of recruitment, psychotherapy training institutes throughout Germany were contacted (see Additional file 1 for currently cooperating institutes). Inclusion criteria that institutes must fulfill are: (1) offering training in cognitive behavioral therapy and/or psychodynamic therapy for adults and/or for children and adolescents, (2) recognizing the workshops as a part of their curriculum. As the workshops will be conducted in an online setting, there is no restriction regarding the size of the institutes. All recruitment steps undertaken to recruit the trainee sample were arranged closely with the cooperating institutes. This included, e.g., the distribution of information material via e-mail or the organization of information events.

Regarding the trainee sample, individuals of all genders and ages will be included if they fulfill the following inclusion criteria: (1) being in training for cognitive-behavioral therapy or psychodynamic therapy for adults and/or adolescents, (2) having already started with supervised outpatient treatment, (3) having about five patients (fulfilling the criteria below) they will see for another 24 weeks in the outpatient setting.

Concerning the patients included in the study by their therapist (trainees), the following inclusion criteria apply (1) treatment in an outpatient setting, (2) patients, that are treated by child and adolescent therapists, must be at least 18 years old.

### Procedure

Interested trainees of all cooperating institutes will enroll to the study themselves by initially contacting the project members. Afterwards, they will be randomized by an assigned project member in groups of 36 (cluster size) in a 1:1:1:1 ratio to the three intervention groups and the control group. Randomization will be conducted via an online random-number-generator [52]. Since randomization is performed across all cooperating institutes, possible institute-effects can be eliminated. Blinding of the study team and workshop conductors is not possible due to the nature of the interventions. However, blinding of the FIS video rating is ensured by the use of filters to make the participants’ faces unrecognizable. Trainees as well as patients are fully blinded throughout the study. Data will be electronically gathered via the online-platform SoSci Survey and managed by the data coordinators (AB and StS). In case of the FIS ratings, these will first be created manually by the raters (AB and StS), then imported into the data-set and finally double checked by a project member not involved in the rating process. Informed consent will be obtained prior to the first assessment virtually (regarding questionnaires for trainees and patients) as well as through signature (regarding image and sound-recording for trainees)^1^. To protect the confidentiality of participants, all personal and assessment data is stored exclusively on university servers. The trainees receive an allowance of 100€ for full participation in the survey which will be transferred in two stages (50€ after post-survey and 50€ after follow-up). In addition, the trainees can have the intervention counted as theory units within their training. After finishing the post-survey, trainees will receive an invitation by email to participate in video call interviews, outlining their purpose and procedure. Drop-outs will be invited to participate as interviewee as well. Interviews will be undertaken with trainees of all four intervention groups, balancing sampling across the groups. The sub-sample is aimed to obtain as much variation as possible by trainees’ practice experience as a therapist, their therapy specialization (cognitive behavioral therapy or psychodynamic therapy) and their patients’ age group (adults or children and adolescents). The sample’s heterogeneity intends to generate a wide variation of perspectives and to reduce premature generalizations. Recruitment will be an ongoing process until the planned number of interviewees is reached (see statistical analyses and sample size). One-on-one interviews will be conducted by WA through a video call platform, compliant to data privacy. WA is a psychotherapist (cognitive behavioral therapy), familiar with the context of psychotherapy training, and exclusively undertaking the qualitative research. At the beginning of the interview, the purpose and procedure will be clarified and explained to the trainee. Permission to audio record the interview and a written consent form will be obtained ^1^. A pilot tested, semi-structured interview script will be used to ensure uniformity, based on literature review and objectives of the qualitative component. All interviews will be audio-recorded, transcribed verbatim, and anonymized.

### Intervention

The intervention is carried out in form of three consecutive workshops which are offered as a part of the theoretical curriculum of the cooperating psychotherapy institutes. All participants, irrespective of their group allocation, will receive the same duration of teaching units (dosage). By using an active control group, it will be possible to relate the difference in the learning progress to the respective didactic method instead of the engagement with and time investment in the subject of interpersonal skills. In terms of structure/organization and content/theoretical framework, the interventions do not differ neither between the intervention groups nor to the active control group. Irrespective of the group assignment, the workshop group size will range from six to nine participants. Each workshop will be facilitated by one project employee and one licensed supervisor^2^. Each teaching unit lasts four hours, of which approximately 1.5 h are scheduled for theoretical input and 2.5 h for the practical exercise. All workshops will take place in an online setting via Zoom, utilizing breakout sessions for the exercise parts. A clear distinction between the groups is not apparent until the practical exercise part of the workshops.

The theoretical framework of the workshops is based on an integration of the AFT [37] and the FIS [24]. Conceptually, both approaches target to improve the therapist’s relational skills and thus strengthen the therapeutic alliance. In line, the primary aim is to create an awareness for subliminal reactions and therapeutic, interpersonal processes in order to generate more empathic and relationship-oriented reactions in therapy. For the teaching units, the concepts of the AFT are used as a theoretical basis. In this context, the trainees will be introduced to the basic concepts of therapeutic alliance, alliance ruptures and repair strategies [53, 54]. In addition, key skills such as self-awareness and interpersonal sensitivity will be taught. The components of the FIS, in the form of the eight subcategories, will also be covered in theory and taught to the trainees. Due to the segmentation of the content into three workshops, the subjects are bundled accordingly and thus form the different foci of the respective workshops, namely *introduction of basic skills*,* withdrawal ruptures*, and *confrontation ruptures.* A detailed presentation of the workshops’ content can be obtained from Additional file 2.

The conceptual differences between the intervention groups and the control group become apparent during the practical exercise part of the workshops. Within this process, two different didactic methods (Deliberate Practice and Structured Feedback) are applied to practice the content conveyed in the theoretical part. It is important to notice that the training scenes, which are inspired by the FIS Performance Task and used for the practical exercise, are pre-scripted and therefore identical for all intervention groups and the control group. Next, the implementation of the didactic tools will be explained before illustrating the composition of the intervention groups and control group.

Deliberate Practice is characterized by a high rate of repetitions of the same goal-oriented trial focusing on a small part of a more complex task [40]. Within this study, Deliberate Practice is conducted through the work with simulation patients. Prior to the scene being started, target criteria (based on the repair strategies) are defined to guide the trainees’ reactions. The simulation patients then perform the prescribed scene, to which the trainees react in accordance with the target criteria. After the simulation scene has been played out, the workshop instructors provide expert feedback on the implementation of the target criteria. As Deliberate Practice should always be a challenging but not overwhelming exercise, there is the option to adjust the difficulty before the next round. This loop will be repeated a couple of times [54].

Structured Feedback will be based on seven out of eight facets of the FIS, namely verbal fluency, hope and positive expectations, persuasiveness, emotional expression, warmth, acceptance and understanding, empathy, and alliance bond capacity. The eighth scale alliance rupture-repair responsiveness will be replaced by the withdrawal, confrontation, and repair items of the Rupture Resolution Rating System (3-RS; [55]). The purpose for this change is due to the more detailed rupture-repair information delivered by the 3-RS compared to the eighth FIS scale. The Structured Feedback sheet can be obtained from Additional File 3.

As mentioned above, this study will evaluate the effects of each of the didactic tools as well as their combination. Consequently, the following intervention groups can be identified:

Intervention group 1: This group will be trained using Deliberate Practice and Structured Feedback. Accordingly, the trainees will train through the simulation of complex therapy situations with simulation patients. Additionally to the feedback regarding the Deliberate Practice target criteria, the trainees will receive a Structured Feedback concerning their interpersonal skills and repair-behavior.

Intervention group 2: This group will be trained using only Deliberate Practice. Therefore, the trainees will only train through the simulation of ruptures with simulation patients. There will be feedback regarding the target criteria but no Structured Feedback concerning their interpersonal skills.

Intervention group 3: This group will be trained using only Structured Feedback. Thus, the trainees will watch short-videos of the same complex therapy situations used in the intervention groups 1 and 2. Analogously to the FIS Performance Task, those videos contain monologues of patients which the trainees have to reply to verbally as if they were their therapist. Afterwards, they will receive feedback regarding their interpersonal skills and repair-behavior. There will be none or a maximum of one repetition of the same scene per trainee.

Control group: This group will not actively practice the reaction to complex therapy situations. Instead, they will watch the same videos used in intervention group 3 and analyze the scenes according to the rupture situation and possible repair strategies within the group.

Irrespective of the specific intervention, the trainees will continue to participate in the regular curriculum of their training institutes. The changes in experience and learning processes due to the curriculum are assessed as a secondary outcome and thus monitored. As the intervention was designed on the basis of empowerment and is therefore not deficit-oriented, it can be assumed that no side- or unintended effects will occur. In the unlikely event that unintended effects appear, the trainees have the opportunity to express this during the workshops or to contact the seminar instructors individually and discuss ways of dealing with these effects. Patients on the other hand can contact the study members via e-mail in an unlikely event of side effects. Furthermore, a quality control of the seminar will be carried out in which, among other things, the practical benefits of the seminar content for the trainees’ everyday work-life and its implementation will be assessed.

## Measures

### Trainee sample

#### Primary outcome

##### Facilitative interpersonal skills Performance-Task

The Facilitative Interpersonal Skills Performance-Task (FIS Performance Task; [20]) is a method to assess the interpersonal competencies of psychotherapists. Participants have to react to eight short videos depicting difficult therapy-situations. The reaction is captured on video and rated on eight dimensions of interpersonal competencies (verbal fluency; hope and positive expectations; persuasiveness; emotional expression; warmth, acceptance and understanding; empathy; alliance bond capacity; alliance rupture-repair responsiveness) that can be summed up in an overall-score. The videos are coded on a 5-point Likert-scale, ranging from *not characteristic* *(1)* to *extremely characteristic* (5). A recent meta-analysis confirmed the inter-rater reliability for the FIS Performance-Task as high with an intra-class correlation (ICC) ranging from 0.8 to 0.95 with only two exceptions (0.61 and 0.69) [22]. Additionally, investigations show good ecological and content validity [22]. Preparing this project, the in-house translated German version was used and will be validated. Within this study, all videos will be rated by two reliable raters (AB and StS) in order to assess the inter-rater-reliability.

#### Secondary outcomes

##### Brief form of the interpersonal competence questionnaire

The Brief Form of the Interpersonal Competence Questionnaire (ICQ-15; [56]) is a self-report measure containing 15 items to assess social competence via five distinct, related subscales (initiation, negative assertion, emotional support, disclosure, and conflict management). The items are rated on a 5-point Likert-scale ranging from *I am poor at this; I’d feel so uncomfortable and unable to handle this situation*,* I’d avoid it if possible* [1] to I*’m extremely good at this; I’d feel very comfortable and could handle this situation very well* [5]. A German validation study confirmed the internal consistency of the German version to be high (*α* = .87) as well as its factor structure to be good (TLI = .950; CFI = .962; RMSEA = .044) [56].

##### Trainee background information form and trainee current practice report

The Trainee Background Information Form (TBIF) and Trainee Current Practice Report (TCPR) are measures developed by the Society for Psychotherapy Research Interest Section on Therapist Training and Development (SPRISTAD) and are based on the Development of Psychotherapists Common Core Questionnaire (DPCCQ; [57]). The TBIF allows the collection of data concerning demographics, training program information, the professional and personal background, relational manner, and motivation for becoming a psychotherapist [58]. The TCPR assesses the practice experience of the therapists, their treatment settings, how they picture themselves as therapists, current supervision, their current experience in the training program, helpful and hindering training experiences, and questions regarding their present living-situation. The questionnaires consist of different types of questions such as multiple choice, Likert-scales and numeric questions, which is why the reliability and validity cannot be evaluated.

##### Deliberate practice attitudes

The Deliberate Practice Attitudes (DBA; [59]) is an unpublished self-report questionnaire by Blümel and colleagues to assess attitudes toward Deliberate Practice with a length of 13 items. Each item is rated on a 5-point Likert-scale from *totally disagree* (1) to *totally agree* (5). The items can be categorized into three subscales (critical self-reflection and feedback, overcoming hurdles and putting in the effort, and ambition and strive for excellence). Further validation will take place in the course of this project.

#### Process variables

##### Mentalizing emotions questionnaire

The Mentalizing Emotions Questionnaire (MEQ; [60]) is a 16-item self-report questionnaire capturing different facets of the ability to mentalize emotions. The instrument consists of three subscales (self, communicating, and others) that can be summed up in an overall-score. The score for each subscale is generated by the mean of its underlying items which are answered on a 7-point Likert-scale ranging from *never* (1) to *always* (7). Initial investigations on reliability (α_overall−score_ = .95) and construct validity show good results [60].

##### Experiences in close relationships

Adult attachment is measured with the German short-form of the Experiences in Close Relationships (ECR-RD12; [61]). The questionnaire consists of 12 items whose answers are given on a 7-point Likert-scale ranging from *total disagreement* (1) to *total agreement* (7) and is recommended to be used as a screening measure of attachment styles in primary care. Attachment is measured on the dimensions anxiety and avoidance which are calculated using the mean of the corresponding items. The internal consistencies for these scales are within an adequate range (α_anxiety_ = .88; α_avoidance_ = .87) and there are indications for construct validity [61].

#### Additional assessment

##### Satisfaction regarding the workshops

In order to assess the participants’ satisfaction with the workshops, we generated an item pool with seven items, based on the questionnaire for satisfaction with the stationary care (ZUF; [62]). The subjective overall quality, the felt relevance and the didactic quality is measured using a 6-point Likert-scale ranging from *very good* (1) to *inadequate* (6). Furthermore, four open questions are included in the evaluation, in which negative and positive aspects of the training, suggestions for improvement as well as other feedback are explored.

##### Semi-structured interviews

Problem-centered interview [63] scripts will be designed to encourage interviewees to explore and display their experiences during and after the training. The script will be developed based on guidelines suggested by Helfferich [64]. Questions will cover the following areas: initial expectations of the training, perspectives on the training’s impact and significance, subjective views on facilitators and barriers to use as well as implementation. In the end of the interviews, participants will have the chance to express additional aspects. The interviews will be scheduled to last approximately 45 to 60 min.

### Patient sample

#### Primary outcome

##### Depression anxiety stress scales

The Depression Anxiety Stress Scales in its German short form (DASS-21; [65]) is a screening measure for symptoms of depressions, anxiety, and stress consisting out of 21 items. The rating of the symptoms’ burden within the last week is done on a 4-point Likert-scale ranging from *Not at all* (0) to *Most of the time* (3). It results in three distinct scales (depression, anxiety, and stress) calculating the sum of the items. Validation studies show moderate to good results regarding internal consistency (α_anxiety_ = .80; α_stress_ = .87; α_depression_ = .88) as well as high convergent validity [65].

#### Secondary outcome

##### Inventory of interpersonal problems

The Inventory of Interpersonal Problems (IIP-32; [66]) is a self-report questionnaire for assessing interpersonal difficulties. It is a short form of the Inventory of Interpersonal Problems (IIP; [67]), containing 32 items. The IIP-32 is based on the interpersonal circumplex model, describing interpersonal behavior on the two dimensions *affiliation* and *dominance* [68] and assesses those using four subscales for each dimension (self-assurance, assertion, self-reference, withdrawnness, submission, altruism, harmony, and social acceptability). The rating is done on a 5-point Likert-scale ranging from *Not at all* (0) to *Very* (4). For each subscale the sum of the corresponding items is calculated. The overall score is the mean of the eight subscales. The internal consistencies of the subscales have an average of α_mean_ = .79 and there is evidence for the convergent and discriminant validity as well as the circumplex-structure of the scales [66].

#### Process variables

##### Working alliance inventory

The Working Alliance Inventory – short revised (WAI-SR; [69]) is used to assess the therapeutic alliance between the patient and therapist. Based on Bordin’s theoretical model [26], it consists of three subscales: bond, tasks, and goals, each represented by the mean of four items. The items are measured using a 5-point Likert-scale ranging from *rarely* (1) to *always* (5). The overall score is calculated by using the mean of the subscales. The internal consistency is high for all subscales (.82 ≤ α_bond_ ≤ .83; .85 ≤ α_tasks_ ≤ .86; .81 ≤ α_goals_ ≤ .91) and results are indicating good convergent validity [69].

##### Rupture-Resolution-Rating-System Self-Report

The Rupture-Resolution-Rating System Self-Report (3-RS-SR; [55]) is a self-report questionnaire, based on the Rupture Resolution Rating System (3-RS; [55]), to assess rupture-situations and repair-behavior in the therapeutic alliance. The patients’ internal experience is assessed by a list of possible rupture-situations which could have occurred during the therapy session. Rupture-situations that were experienced are rated on a 7-point Likert-scale ranging from *No negative impact at all* (1) to *Extreme negative impact* (7), regarding the negative impact of those situations on their overall ability to work together with their therapist. The same procedure applies to withdrawal- and confrontation behavior on both, the patient’s and the therapist’s side. Furthermore, the questionnaire assesses the patient’s perception of how well they and their therapist worked together during the last session. Regarding this topic, there are six items that are rated on a 7-point Likert-scale ranging from *Not at all* (1) to *A lot/Extremely* (7). The same procedure applies to two items asking for possible repair behaviors shown by the therapist. Due to the novelty of the questionnaire, no validation studies have yet been carried out. The validation of the German version is a part of this study.

The MEQ and the ECR-RD12 are used in the patient sample in the same way as described in the trainee sample.

### Trained supervisor sample

#### Adherence-checklist

In order to assess the implementation fidelity and adherence of our trainings, we generated items based on Gerstner and Finney [70]. The checklist is completed by the co-lecturers and includes questions about their previous lecturing experience as well as a rating regarding the quality of the implementation of the didactic methods and the trainees’ reaction to the didactic methods. The questionnaire ends with an overall rating of the quality, relevance, and didactic quality of the session followed by four open questions regarding positive and negative aspects, suggestions for improvement, and further feedback. All ratings were done on a 5-point Likert-scale ranging from l*ow* (1) to *high* (5).

**Table 1:** Schedule of enrolment, allocation, and assessments

	Trainees						Patients					
Timepoint	t_− 1_	t_0_	t_1_	t_2_	t_3_	t_4_	t_− 1_	t_0_	t_x_^a^	t_y_^b^	t_2_	t_3_
**Enrolment**:												
Eligibility Screen	X						X					
Informed consent	X						X					
Allocation	X											
**Interventions**:												
Workshop 1*		X										
Workshop 2*				X								
Workshop 3 *					X							
**Assessments**:												
**Primary Outcome**:												
FIS		X		X	X	X						
DASS								X		X	X	X
**Secondary Outcome**:												
TBIF/TCPR		X		X	X	X						
ICQ-15		X		X	X	X						
IIP-32								X		X	X	X
DPA		X			X							
**Process Variables**:												
WAI-SR								X	X		X	
3RS-SR								X	X		X	
MEQ		X		X	X	X		X		X	X	X
ECR-RD 12		X		X	X	X		X			X	
**Additional Assessments**:												
Workshop Evaluation			X	X	X							

Notes. * same time-points for all intervention-groups and control-group; ^a^ after every session; ^b^ after every fifth session

### Statistical analyses and sample size

The required sample size was calculated a priori via G*Power [71]. The analysis was based on a variance analytic 4 × 3 design with group assignment (CG vs. IG1 vs. IG2 vs. IG3) as a between-subject factor and time (pre vs. intermediate vs. post) as a within-subject factor. To ensure the possibility of more complex statistical analyses (e.g., multilevel-analyses), we applied a high power of 1 - β = 0.95. Assuming a medium effect size (*f* = 0.20) and a medium correlation between the repeated measures (*r* = .30), the sample size analysis revealed a minimum sample of *N* = 156 trainees. According to the recommendations of Schiefle et al. [72] regarding trainee effects and sample size planning, if each trainee is analyzed with five patients, a sample of *N* = 200 trainees is expected to be sufficient for an average expected effect size of 6.7% and a CI of 4%. Considering a potential drop-out of 20%, *N* = 240 trainees should initially be included in the study. As each trainee will treat at least five patients, there should be *N* = 1000 patients included in the study. Data collection will be stopped after achieving the targeted sample size.

Regarding the qualitative part of the study, the aim is to interview four to six trainees per group (total of 16 to 24 interviews). There will be room for flexibility as data saturation will be currently evaluated during the data collection period. Several open-ended survey questions and semi-structural interviews offer a rich amount of qualitative data. Focusing on the objectives of the embedded qualitative component, the data will be analyzed using qualitative content analysis [73] and thematical analysis [74].

The two samples (trainees and patients) and the large number of both measurement points (four for trainees and 25 for patients) as well as assessed variables result in a large data set. Due to the variety of subprojects and different foci of data analyses, many statistical models will be calculated in order to answer the corresponding research questions for each subproject. Accordingly, the statistical analyses will be examined in more detail in the individual publications and only general approaches and tactics will be highlighted in the following.

As soon as all baseline assessments are finished, cross-sectional analyses will be carried out. These analyses will include questionnaire validations (DPA, MEQ, and 3RS-SR) as well as cluster-analyses and multiple regression analyses. Regarding the longitudinal data, analyses will be performed after the completion of data collection. All timepoints of assessment will be used for analyses to ensure to adequately investigate the within-subject effects. Therefore, multilevel-modelling and structural equation modelling will be conducted. Regarding the frequentist approaches, two-tailed nominal *p*-value = .05 and 95%-confidence intervals will be used. The structural equation models will be evaluated according to their model fit indices (Comparative Fit Index, Root Mean Square Error of Approximation, and Standardized Root Mean Square Residuals). In addition to calculating the group differences within the RCT, the reliable change index will be used to quantify significant intra-individual changes with regard to the FIS. Missing values will be replaced using multiple imputation. All analyses will be performed using R Studio [75].

## Discussion

This study uses an RCT to investigate the efficacy of Deliberate Practice, Structured Feedback and their combination in teaching interpersonal competencies to trainees in a psychotherapy training context. Furthermore, the study also examines whether an increase in interpersonal skills among trainees has an impact on the quality of their outpatient therapies. Although individual effects of therapy-relevant components have already been shown in previous studies (e.g., efficacy of Deliberate Practice [42] or the relationship between interpersonal skills and perceived therapeutic alliance [32]), no study has yet investigated this multitude of variables in an overarching approach in both a trainee and patient sample. The results of the study are intended to contribute to improving the effectiveness and focus of psychotherapy training and to implementing new learning methods. The overarching aim is therefore to enhance the competencies of future therapists and thus improve the quality of future therapies.

In the course of this, some strengths of the study can be recognized. Initially, the longitudinal RCT design with an active control group can be mentioned as a strength. Together with the fact that both the therapist variables and the patient/therapy variables are included in the study, the survey offers an overarching and comprehensive view of the therapeutic relationship process. In addition, the data will be collected from trainees of different institutes and specializations (CBT and PT), so as to enable a high degree of generalization of the results. Interviews will supplement the quantitative measures, allowing trainees’ perspectives to be included, and be vital in generating hypotheses for training implementation and future studies. Lastly, the combination of two already established didactic methods and the comparison with the individual use of those methods should be highlighted as an advantage.

Despite the strengths, some limitations need to be addressed. For example, it should be noted that the seminar instructors cannot be blinded with regard to the corresponding intervention group and resulting effects cannot be controlled accordingly. Furthermore, the voluntary nature of the patients’ participation could lead to patients participating who have a better relationship with the therapists from the outset, thus distorting the results. Finally, it should be mentioned that the implementation of an active control group precludes checking whether the purely theoretical engagement with the topic of competencies and alliance (as is done in the active control group) leads to an improvement in the FIS. This needs to be investigated in further projects. In conclusion, the study offers the opportunity to close an important research gap in the field of psychotherapy training and thus will contribute to improving the training and assuring the quality of future psychotherapies.

## Electronic supplementary material

Below is the link to the electronic supplementary material.

Additional file 1: Appendix A. List of participating institutes (Status June 2024).

Additional file 2: Appendix B. Theoretical and practical content of the workshops.

Additional file 3: Appendix C. Structured Feedback Evaluation Sheet.

## Data Availability

No datasets were generated or analyzed during the current study.

## Footnotes

1 If interested, informed consent material can be obtained from the authors.

2 All supervisors were given a 3.5-hour training course on how to implement the didactic tools. The training was conducted under the direction of the project members.

## Abbreviations

3-RS: Rupture Resolution Rating System
3-RS-SR: Rupture-Resolution-Rating System Self-Report
AFT: Alliance-Focused Training
CBT: Cognitive Behavioral Therapy
DASS-21: Depression Anxiety Stress Scales, German short-form
DBA: Deliberate Practice Attitudes
DeeP: Deliberate Practice and Structured Feedback in Psychotherapy training
DFG: Deutsche Forschungsgemeinschaft – German Research Foundation
DPCCQ: Development of Psychotherapists Common Core Questionnaire
DRKS: Deutsches Register Klinischer Studien
ECR-RD12: Experiences in Close Relationships, German short-form
FIS: Facilitative Interpersonal Skills
ICC: Intra-class Correlation
ICQ-15: The Brief Form of the Interpersonal Competence Questionnaire
IIP-32: Inventory of Interpersonal Problems, short-form
MEQ: Mentalizing Emotions Questionnaire
PT: Psychodynamic Therapy
RCT: Randomized-Control-Trial
SPRISTAD: Society for Psychotherapy Research Interest Section on Therapist Training and Development
TBIF: Trainee Background Information Form
TCPR: Trainee Current Practise Report
WAI-SR: Working Alliance Inventory – short revised
