# Supplementary material for: Effects of deliberate practice and structured feedback in psychotherapy training (DeeP): a study protocol of a randomized-control-trial
Source: BMC Psychol. 2024 Dec 4;12:719. doi: 10.1186/s40359-024-02015-x (PMC11616299; doi:10.1186/s40359-024-02015-x)
Supplement: Supplementary file 1 — Additional file 1: Appendix A. List of participating institutes (Status June 2024). [file 40359_2024_2015_MOESM1_ESM.pdf]

Appendix A. List of participating institutes (Status June 2024)

**Name**	**Location**	**Training**	**Homepage**
Akademie für Psychotherapie	Erfurt	CBT and PT	https://www.afp-erfurt.de/
Akademie für angewandte Psychologie und Psychotherapie	Köln	CBT and PT	https://www.app-koeln.de/
Centrum für Integrative Psychotherapie	Bamberg/Erlangen	CBT	https://cip-bamberg.de/
Institut für Fort- und Weiterbildung in Klinischer Verhaltenstherapie	Bad Dürkheim	CBT	https://www.ifkv.de/
Köln-Bonner Akademie für Psychotherapie	Köln/Bonn	PT	https://www.kbap.de/
Köln-Bonner Akademie für Verhaltenstherapie	Köln/Bonn	CBT	https://www.kbav.de/
Magdeburger Ausbildungsinstitut für Psychotherapeutische Psychologie	Magdeburg	CBT and PT	https://www.mapp-institut.de/
Münchner Arbeitsgemeinschaft für Psychoanalyse	München	PT	https://www.psychoanalyse-map.de/
Instituts für Psychotherapie am Universitätsklinikum Hamburg-Eppendorf	Hamburg	CBT and PT	https://www.uke.de/kliniken-institute/institute/institut-fuer-psychotherapie/index.html
Zentrum Ausbildung Psychotherapie	Bad Salzuflen	CBT and PT	https://zap-lehrinstitut.de/
Zentrum für Psychotherapie	Münster	CBT	https://www.zpt-muenster.de/
Institut für Verhaltenstherapie-Ausbildung Hamburg	Hamburg	CBT	https://www.ivah.de

Notes. CBT = cognitive behavioral therapy, PT = psychodynamic therapy
