# Supplementary material for: Effects of deliberate practice and structured feedback in psychotherapy training (DeeP): a study protocol of a randomized-control-trial
Source: BMC Psychol. 2024 Dec 4;12:719. doi: 10.1186/s40359-024-02015-x (PMC11616299; doi:10.1186/s40359-024-02015-x)
Supplement: Supplementary file 2 — Additional file 2: Appendix B. Theoretical and practical content of the workshops. [file 40359_2024_2015_MOESM2_ESM.pdf]

Appendix C. Structured Feedback Evaluation Sheet

Verbal Fluency

1	2	3	4	5
Difficulty verbalizing a response (avoidance or anxiety)	Rather anxious verbal expression; choppy, halting communication	Moderate level of verbal fluency, conversational, mostly easy to follow	Fluent response; little that is difficult to follow	Approaching, relaxed, melodic, rhythmical and easy to follow

Hope and Positive Expectations

1	2	3	4	5
Hopeless or pessimistic	Some hopelessness; feeling unable to help the patient	Optimism not directly perceptible, hopefulness is not expressed convincingly	General sense of optimism; addressing the client's agency	Hopeful, addressing the client's agency (inclusion of the client themselves)

Persuasiveness

1	2	3	4	5
Unorganized, incoherent, and difficult to follow	Unpersuasive (lack of credibility or lack of persuasiveness)	Little sense of persuasiveness	Persuasive; rather implicit rationale, possibly unclear or marginally relevant	Highly persuasive; re-framing of the client's experience

Emotional Expression

1	2	3	4	5
Little/no emotional expression	Display of Interest/curiosity, but little emotional expression	Emotional expression similar to ordinary conversation	Appropriate level of emotional expression (less focused)	Vocal expression is highly emotional (but not primarily negative)

Warmth, Acceptance & Understanding

1	2	3	4	5
Obvious lack of respect, acceptance and warmth	Subtle lack of respect, acceptance or concern	Ordinary level of courtesy and warmth or opinion of the client may not be clearly discernable	Nonjudgmental and interested in the client's thoughts and feelings. Caring and respectful	Obvious expression of warmth, concern and acceptance

Empathy

1	2	3	4	5
Clear distortion of the client's experience and misidentification of complaints, beliefs, emotions etc.	No awareness or understanding of the client's experience, possibly minor distortions	Accurate commenting about obvious aspects of the client's experience	Accurate but less intense commenting on the client's experiences	Expression of an extraordinary understanding of the client's experience (including the client's non-verbal expression)

Alliance Bond Capacity

1	2	3	4	5
Actively undermining a mutual collaboration	Slightly undermining a collaborative atmosphere (may be unintentionally)	Neither undermining nor enhancing a collaboration	Efforts for collaboration	Creating a collaborative atmosphere; Creating a "we-ness"

Evaluation criteria 3Rs – is only mentioned in case of actual occurrence

Criterion	Not very salient		Somewhat salient		Very salient
Therapist withdraws (shuts down, avoids, masks their real experience)					
Therapist confronts (complaints/criticizes, pushes back, controls/exerts pressure)					
Therapist focuses on the task/the goal					
Therapist explores the rupture					
Therapist acknowledges own contribution to rupture					
