# Supplementary material for: Effects of deliberate practice and structured feedback in psychotherapy training (DeeP): a study protocol of a randomized-control-trial
Source: BMC Psychol. 2024 Dec 4;12:719. doi: 10.1186/s40359-024-02015-x (PMC11616299; doi:10.1186/s40359-024-02015-x)
Supplement: Supplementary file 3 — Additional file 3: Appendix C. Structured Feedback Evaluation Sheet. [file 40359_2024_2015_MOESM3_ESM.pdf]

Appendix B. Theoretical and practical content of the workshops

	**Workshop 1**<br>Introduction of basic skills	**Workshop 2**<br>Withdrawal ruptures	**Workshop 3**<br>Confrontation ruptures
Introduction (10min)	Social gathering and introduction of the study	Exchange of experiences and further feedback	Exchange of experiences and further feedback
Theory (50min)	- Alliance [26]<br>- Interpersonal competencies [20]<br>- Concept of ruptures [53]<br>- Repair-strategies (55)<br>• Validation<br>• Exploration<br>• Making a process comment<br>- Exemplary video and discussion	- Withdrawal ruptures [53]<br>• Interpersonal markers<br>• Intrapersonal markers<br>- Repair-strategy [53]<br>• Negotiating tasks and goals<br>- Exemplary video and discussion	- Confrontation ruptures [53]<br>• Interpersonal markers<br>• Intrapersonal markers<br>- Repair-strategy [34]<br>• Finding interpersonal pattern<br>- Exemplary video and discussion
Break (20min)			
Practice (150min)	- Awareness-exercise (5min)<br>- Practice of basic skills<br>• Didactic method is depending on the intervention group	- Awareness-exercise (5min)<br>- Practice of dealing with withdrawal ruptures<br>• Didactic method is depending on the intervention group	- Awareness-exercise (5min)<br>- Practice of dealing with confrontation ruptures<br>• Didactic method is depending on the intervention group
Check-Out (10min)	- Feedback<br>- Organizational matters	- Feedback<br>- Organizational matters	- Feedback<br>- Organizational matters

Notes. Introduced repair-strategies in the respective workshops are not meant to be constraint to the rupture type taught in the same workshop
